# The relations between Czech undergraduates’ motivation and emotion in self-regulated learning, learning engagement, and academic success in blended course designs: Consistency between theory-driven and data-driven approaches

**DOI:** 10.3389/fpsyg.2022.1001202

**Published:** 2022-11-18

**Authors:** Feifei Han, Jitka Vaculíková, Kateřina Juklová

**Affiliations:** ^1^Institute for Learning Sciences and Teacher Education, Australian Catholic University, Brisbane, QLD, Australia; ^2^Department of Pedagogy and Psychology, Faculty of Education, University of Hradec Králové, Hradec Králové, Czechia; ^3^Research Centre of FHS, Faculty of Humanities, Tomas Bata University in Zlín, Zlín, Czechia

**Keywords:** self-regulated learning, self-reported measures, observational measures, academic success, blended course designs, Czech Republic

## Abstract

Combining theory-driven and data-driven approaches, this study used both self-reported and observational measures to examine: (1) the joint contributions of students’ self-reported undergraduates’ motivation and emotion in their self-regulated learning, their observed online learning interactions, and their academic success in blended course designs; and (2) the extent to which the self-reported and observational measures were consistent with each other. The participants in the study were 54 social sciences undergraduates in the Czech Republic. The participants’ self-reported self-efficacy, intrinsic goals, and anxiety were assessed using a Czech version of three scales from the Motivated Strategies for Learning Questionnaire. Their online engagement was represented by students’ observed frequency of interactions with the six online learning activities recorded in the learning management system. The results of a hierarchical regression analysis showed that the self-reported and observational measures together could explain 71% of variance in academic success, significantly improving explanatory power over using self-reported measures alone. Departing from the theory-driven approach, students were clustered as better and poorer self-regulated learners by their self-reports, and one-way ANOVAs showed that better self-regulated learners had significantly more frequent online interactions with four out of six online learning activities and better final exam results. Departing from the data-driven approach, students were clustered as higher and lower online-engaged learners by the observed frequency of their interaction with online learning activities. One-way ANOVAs showed that higher online-engaged learners also reported having higher self-efficacy and lower anxiety. Furthermore, the strong association between the students’ profiles in both self-reported measures and observational measures in cross-tabulation analyses showed that the majority of better self-regulated learners by self-reporting also had higher online engagement by observation, whereas the majority of poorer self-regulated learners by self-reporting were lower online-engaged learners, demonstrating consistency between theory-driven and data-driven approaches.

## Introduction

Advances in Internet- and computer-based technologies have increased the growth of alternative learning spaces, creating entirely new ways of conceptualizing and assessing the learning experience of students (Wong, 2022). Research has repeatedly found positive impacts of various forms of technology-enhanced learning, such as those embedded in online tutorials, social networking sites, computer-supported collaborative learning, web-conferencing, webinars, e-portfolios, digital games, simulations, and virtual worlds (Jarvoll, 2018; Winkelmann et al., 2020). The higher education sector has shown rapid development in the use of computer- and web-based technologies, which has affected the learning experience of a significant proportion of university students worldwide. This change has resulted in the widespread use of diverse online spaces developed for learning in the form of e-learning courses and combinations of face-to-face and online delivery modes, known as blended course designs (Pardo et al., 2016a; Shin et al., 2018).

More recently, the coronavirus (COVID-19) pandemic emergency has required higher education learning and teaching around the world to rapidly respond and, in particular, to redeploy even more learning and teaching activities to virtual learning spaces to promote physical distancing. As a result, vast numbers of face-to-face courses have been delivered either as blended courses or as purely online courses (Tang et al., 2021). Learning in online and/or blended contexts requires students to take high levels of control of and to regulate their learning (Vanslambrouck et al., 2019; Wong et al., 2019). This has resulted in an increasing number of studies examining students’ self-regulated learning in online and blended deliveries (Broadbent and Fuller-Tyszkiewicz, 2018; Sun et al., 2018). These studies have focused on the various computer-generated scaffolds and characteristics of online learning tasks, which may affect students’ self-regulated learning behaviors (Winters et al., 2008; Dabbagh and Kitsantas, 2013; Venkatesh et al., 2013), and how different aspects of self-regulated learning, such as motivation, emotion, and effort, impact students’ academic success (Li et al., 2020).

To investigate how different elements in self-regulated learning are related to academic success, for quite a long time, research has predominantly adopted theory-driven approaches, which test hypotheses derived from theories in educational psychology, the learning sciences, and pedagogy and curriculum research through self-reported instruments and measures (Han, 2022). In recent years, the modern development of learning analytics generated by advanced algorithms makes it possible to monitor and record students’ online learning behaviors through the digital trace data left in online learning management systems (LMS; Ainley and Patrick, 2006). As a result, an increasing number of studies have departed from this digital trace data to understand students’ self-regulated learning, known as data-driven approaches (Barnard-Brak et al., 2011; Dent and Koenka, 2016; Martinez-Maldonado et al., 2016; Pardo et al., 2016a). However, the extent to which theory-driven and data-driven approaches are consistent with each other in terms of understanding students’ self-regulated learning needs to be empirically investigated. The current study combined theory-driven and data-driven approaches to examine Czech undergraduate students’ self-regulated learning experience in blended course designs.

## Theoretical framework

### Social-cognitive perspective of self-regulated learning

Self-regulated learning describes learners’ cognitively and metacognitively oriented thoughts, feelings, and actions toward the attainment of certain learning goals (Zimmerman and Schunk, 2001). Various models have been proposed to describe and conceptualize self-regulated learning (Moos and Stewart, 2013). Some models conceive self-regulated learning as an event-based phenomenon in specific contexts (Greene and Azevedo, 2010; Järvelä and Hadwin, 2013); whereas others describe the overall process of self-regulated learning (Winne and Hadwin, 1998).

The self-regulated models also have different theoretical bases. For instance, Winne and Hadwin’s (1998) model adopts an information processing perspective; the model proposed by McCaslin and Hickey (2001) departs from a sociocultural point of view; and Pintrich’s self-regulated model is based on social-cognitive theory (Pintrich, 2000). Researchers have also attached importance to different aspects in the process of self-regulated learning. While some researchers emphasize metacognitive monitoring and control in self-regulated learning (Winne and Perry, 2000; Winne, 2001), some focus on emotions (Boekaerts and Cascallar, 2006), and others pay attention to the cognitive aspect of self-regulated learning (Winne, 1996).

Although self-regulated learning has been conceptualized differently (Moos and Stewart, 2013), all the theoretical models of self-regulated learning share the following four common underlying assumptions (Pintrich, 2000). First, students should play an active role in constructing meaning from their prior knowledge and the context of their learning. Second, students are able to monitor and regulate their own cognition and motivation. Third, students have the ability to modify their cognition and motivation to achieve their set learning goals. Last, learning is shaped by the interaction between students’ learning contexts and their own characteristics. All the theoretical models share the autonomous and self-directed nature of the self-regulated learning process, but they differ on the importance they attach to various elements (Azevedo et al., 2008).

Among different conceptualized models of self-regulated learning, Pintrich’s (2000) social-cognitive model comprehensively describes the main process of self-regulated learning and is one of the most widely adopted theoretical framework to measure self-regulated (Broadbent and Poon, 2015). This model proposes a triadic reciprocal interaction among motivation, the environment, and behavior, in which self-regulated learning is perceived as a “dynamic and contextually bound” phenomenon rather than a static trait of students (Winne, 2001; Duncan et al., 2005). It proposes that learners’ motivation, cognition, and self-regulated learning strategies may vary depending on the contextual features of the learning environment and characteristics of learning tasks (e.g., the nature of the course, its structure, and the learning activities) along with learners’ internal state of mind (e.g., students’ interest and their abilities) (Pintrich and Zusho, 2002). Therefore, the external environment, such as the course designs (including the design of the online course sites) can either facilitate or hamper self-regulated learning processes (Zimmerman and Schunk, 2011), and may, in turn, affect the academic performance (Broadbent and Poon, 2015). In this perspective, able self-regulated learners are often described as having higher self-efficacy to manage and effectively organize study with a minimum of distractions, feeling less anxious, having appropriate learning goals, and adopting appropriate self-regulated learning strategies (Broadbent and Poon, 2015).

### Research on self-regulated learning using theory-driven approaches

To understand students’ self-regulated learning experience, the majority of existing research adopted a theory-driven approach, which tested hypotheses derived from theories through primary means of self-reported measures, in particular, self-reported questionnaires, such as the Motivated Strategies for Learning Questionnaire (MSLQ; Pintrich and De Groot, 1991). However, the self-reported measures have been criticized for being subjective and have been questioned with regard to their accuracy in describing students’ use of learning approaches and strategies in real learning contexts (Zhou and Winne, 2012). In addition, the self-reported measures and data also suffer from their limited capacity to represent the complex (e.g., using multiple indicators) and dynamic (e.g., changes over time) nature of student learning behaviors. To improve the insights of contemporary university student experiences of learning, suggestions have been put forward to expand the current self-reporting methods by including other types of measures for studying student learning (Vermunt and Donche, 2017). In this regard, learning analytics research is a promising avenue. For instance, Richardson (2017, 359) suggests, “The rapidly expanding field of learning analytics provides both researchers and practitioners with the opportunity to monitor students’ strategic decisions in online environments in minute detail and in real time.”

### Research on self-regulated learning using data-driven approaches

Recent developments in educational technology have produced prolific studies departing from data-driven approaches and have created an area of emerging research in learning analytics. Learning analytics research collects rich, detailed digital traces of students’ interactions with a variety of online learning resources and activities. This type of digital trace/log data, also known as observational data, has the advantage of offering descriptions of students’ online learning behaviors and strategies relatively more objectively and in more granular detail than using self-reported measures (Siemens, 2013; Baker and Siemens, 2016; Sclater et al., 2016). In learning analytics research, the observational data are processed by advanced data mining techniques and algorithms in order to advise students’ career choice (Bettinger and Baker, 2014); to detect at-risk students (Krumm et al., 2014); to provide personalised feedback (Gibson et al., 2017); to facilitate collaborative learning (Kaendler et al., 2015); to monitor students’ affect in learning (Ocumpaugh et al., 2014); to identify patterns of learning tactics and strategies (Chen et al., 2017); and to predict their academic learning outcomes (Romero et al., 2013). For instance, Jovanović et al. (2017) used the 13-week digital trace data extracted from the LMS to investigate 290 computer science students’ self-regulated online learning strategies. The hierarchical sequence analysis identified five distinct groups of students which differed in their self-regulated learning strategies, namely the intensive students (diverse online learning strategies); the strategic students (focusing on summative and formative assessments); the highly strategic students (focusing on summative assessments and reading materials); the selective group (emphasizing summative assessments without much involvement in reading the course materials); and the highly selective group (predominantly performing summative assessments). The students with different self-regulated learning strategies also differed in their academic performance: the first three groups performed significantly better on both mid-term and final exams than the other two groups. Despite presenting informative findings with regard to students’ use of self-regulated learning strategies in online learning, this study failed to reveal information on learners’ internal state of mind, such as their motivation, learning goals, and anxiety, which are important elements in social-cognitive perspectives on self-regulated learning.

This kind of learning analytics research using data-driven approaches has received criticism on the basis of it being split from educational theory and overly focusing on quantitative numbers (Rodríguez-Triana et al., 2015). The patterns and models of students’ learning patterns and strategies derived from such data-centric perspectives without proper guidance from educational theory often result in erroneous interpretation, which has limited insight for generating actionable knowledge in order to locate learning barriers, to offer ideas for teaching practice and curriculum design, and to foster a quality learning experience and to improve academic performance (Buckingham Shum and Crick, 2012; Wong and Li, 2020; Han and Ellis, 2021).

### Research on self-regulated learning by combining theory-driven and data-driven approaches

Recognizing the limitations of the theory-driven and data-driven approaches to researching self-regulated learning, researchers have proposed the adoption of a combined approach when designing research to gain a more comprehensive picture of students’ self-regulated learning. Employing both self-reported and observational measures of students’ self-regulated learning, the combined approach enables investigations of students’ learning to be more holistic by modeling and interpreting big data while being guided *via* sound theories (Lockyer et al., 2013; Gašević et al., 2015; Rienties and Toetenel, 2016).

There is only limited research that adopts a combined approach to investigating students’ self-regulated learning. These existing studies have two different foci. One line of research aims at examining how self-reported and observational measures of students’ self-regulated learning predict their academic access (Reimann et al., 2014). In a study by Pardo et al. (2016a), the first multiple regression analysis which only used self-reported measures (i.e., students’ reported anxiety in learning and their reported use of self-regulated learning strategies) as predictors could only explain 7% of variance in students’ academic performance. In the second multiple regression analysis which added the observational measures (i.e., observed frequency of students’ interactions with online learning activities), an additional of 25% of variance in students’ academic performance was explained. This means that combining self-reported and observational measures could explain 32% of variance in students’ academic performance, much higher than using either self-reported or observational measures alone.

Another focus of the studies which combine self-reported and observational measures is to investigate the extent to which the two types of measures are consistent in describing students’ self-regulated learning. Li et al. (2020), for example, used the observational data collected from an LMS to measure two aspects of students’ self-regulated learning: time management and effort regulation. The results showed that the observational measures were significantly associated with students’ self-reported time management and effort regulation in the post-course survey but not the pre-course survey, demonstrating a certain level of alignment between the more objective observations of what they did to manage time and effort in learning and students’ own interpretations of their time management and effort-regulation behaviors.

Only limited research has addressed the above-mentioned two research aims in a single study In an asynchronous online agriculture course, Ye and Pennisi (2022) investigated: (1) the extent to which the patterns of self-regulated learning measured by self-reported data (i.e., questionnaire and interviews) and observational data (digital traces of students’ online interactions with learning activities, peers, and teaching staff) were consistent; and (2) how the patterns of self-regulated learning measured by two types of data sources contributed to students’ academic success, respectively. With regard to the first research aim, the results showed a certain level of consistency between the self-reported and observational data, as some correlations between the two sets of variables were in an unexpected direction. For instance, students’ self-reported help-seeking was negatively correlated with the average lecture completion rate and the average time spent on additional resources. In addition, students’ self-reported self-evaluation was negatively correlated with the average topics and items visited and lecture completion rate. In accordance with these correlation results, students’ clusters by the self-reported questionnaire and digital trace data showed overlaps were only 30.8% (for the three-cluster solution) and 53.8% (for the two-cluster solution). Concerning the second research aim, two separate sets of regression analyses were conducted to examine how students’ self-regulated learning in the course predicted their final grades. One set of regression analyses used self-reported measures as predictors whereas the other set employed observational measures as predictors. The results found that while the self-reported measure could explain only 11% of variance in students’ final grades; the observational measures could explain as high as 73% of the variance in the final grades. However, the study did not examine how a combination of self-reported and observational measures predict students’ final grades.

The current study will address the above-mentioned two aims in a single study among Czech university students, which is a less researched population in the field of blended learning. The current study had two aims to investigate: (1) the joint contributions of students’ self-reported and observed self-regulated learning; and (2) the extent to which the self-reported and observational measures offer consistent evidence with regard to students’ self-regulated learning. The first aim would be addressed by the first research question, and the second aim would be addressed by the second and third research questions.

(1) What are the joint contributions of students’ self-reported motivational and emotional aspects in self-regulated learning, and how do their observed interactions with online learning activities contribute to their academic success?
(2a) How do observed students’ interactions with online learning activities and academic success differ based on the profiles of students’ self-reported motivational and emotional aspects in self-regulated learning?
(2b) How do students’ self-reported motivational and emotional aspects in self-regulated learning and academic success differ based on the profiles of observed students’ interactions with online learning activities?
(3) To what extent do students’ clusters result from theory-driven and data-driven approaches which are consistent with each other?

## Materials and methods

### The participants and the learning context

The participants were 54 (males = 26; females = 28) full-time second-year university students who were enrolled in a bachelor’s degree program in a middle-sized public university in the Czechia Republic. The mean (*M*) age of the participants was 20.94 (SD = 0.71). The learning context of the study was a research methodology in the social sciences, which was designed using the flipped classroom learning principles. The flipped classroom learning design requires students to engage in “interactive content focusing on key concepts prior to class thus allowing class time for collaborative activities that clarify concepts and contextualise knowledge through application, analysis, and planning and producing solutions” (Karanicolas et al., 2018, p. 1).

For this course, students’ online learning took place online in their own pace and at their own time before and after taking part in the face-to-face classroom learning and teaching. Specifically, before taking face-to-face classroom learning each week, students were required to: (1) viewing instructions on how to navigate the course site (instructions); (2) watching pre-recorded online video lectures (videos); (3) reading compulsory learning materials in either Word documents or PDFs (materials); (4) browsing web pages containing supplementary learning materials (web pages); and (5) answering pre-lecture questions (questions). After taking the in-person classroom learning, students were required to (6) doing test items for the week (tests). The in-person classroom learning was a weekly 2-h lecture each week, which recapped and expanded the concepts from pre-lecture videos; clarified ambiguities and questions from students; and had hands-on practice of data analysis methods with specific examples.

### Measures and instruments

#### Self-reported measures of motivation and emotion in self-regulated learning

The self-reported measures of the motivational and emotional aspects of self-regulated learning were drawn from the MSLQ (Pintrich and De Groot, 1991). The MSLQ consists of two main sections, a motivation section (items 1–31) and a learning strategies section (items 32–81). The 31 items of the motivation section had three scales, namely expectancy (self-efficacy), value (intrinsic goals), and affect (anxiety). However, the translated Czech version of the instrument has been modified according to the Czech educational environment. The modified and validated version (Jakesova and Hrbackova, 2014) had less items in the three similar scales, namely self-efficacy, intrinsic goals, and anxiety. Hence, the three scales were used to measure students’ motivational and emotional aspects of self-regulated learning in this course.

Self-efficacy assessed students’ perceived ability to succeed in the course (6 items, Coefficient *H* = 0.88, e.g., I am confident I can do an excellent job on the assignments and tests in this course). Intrinsic goals measured students’ perceived orientation stemming primarily from internal motivations (3 items, Coefficient *H* = 0.82, e.g., In a class like this, I prefer course material that really challenges me so I can learn new things). Anxiety evaluated students’ perceived feelings of worrying about not performing well in the tests in the course (3 items, Coefficient *H* = 0.80, e.g., When I take a test, I think about how poorly I am doing). All the items were measured on 7-point Likert scales with 1 representing not at all true of me and 7 indicating very true of me.

#### Observational measures students’ interactions with the online learning activities

The observational measures were frequencies students’ interactions with the above-mentioned six online learning activities, which were collected through the LMS—the Modular Object-Oriented Dynamic Learning Environment (Moodle). Although the full functions of Moodle for hosting the course sites were fully enabled for teachers in order to ensure that students have good learning experience, only limited learning analytics functions were released for teachers to monitor students’ learning. Hence, students’ interactions with different online learning activities were only represented in the format of frequency.

#### Measures of academic success

Academic success was measured by the final exam, which consisted of both multiple-choice and open-ended questions, with 5 being the maximum possible score (*M* = 2.22; SD = 0.79).

### Data collection

Prior to the data collection, ethics approval was obtained from the University Human Resources Ethics Committee and the University Institutional Review Board. The participants were informed about voluntary participation and written consent was sought if they indicated a willingness to participate. The questionnaire was distributed online through the LMS and the students were instructed that the answers given on the questionnaire should be a reflection of their learning in this course. Upon the completion of the course, students’ interactions with the online learning activities were drawn from the LMS and their final exam scores were obtained from the course coordinator.

### Data analysis

To answer the first research question—the joint contributions of the self-reported motivational and emotional aspects of self-regulated learning and the observed students’ interactions with online learning activities and academic success—hierarchical regressions were performed using the final exam scores as the dependent variable; the independent variables were decided by using the results of correlation analyses to ensure the linear relationship between independent and dependent variables. The mean scores of the three self-reported scales and the frequencies of the observed students’ interactions with online learning activities which were significantly correlated with the final exam scores were used as independent variables. A number of assumption tests were also conducted. Values of Tolerance and the variance inflation factor were screened to see if there was multicollinearity. The Koenker test was conducted to examine if the homoscedasticity assumption was met. Two models were constructed for hierarchical regression analyses. In the first model, the final exam scores were regressed on the self-reported measures from the questionnaire, as past research has consistently reported that students’ self-efficacy, intrinsic goals, and anxiety in learning contribute to their academic success (Zimmerman, 2008; Beishuizen and Steffens, 2011; Lynn et al., 2011). Based on the first model, the observed students’ interaction with online learning activities was added to examine the extra contribution to academic success made by observational measures.

For the second research question, two separate hierarchical cluster analyses were conducted using the self-reported measures and observational measures, respectively. The first hierarchical cluster analysis used the mean scores of the self-efficacy, intrinsic goals, and anxiety scales to cluster students. On the basis of cluster membership, a series of one-way ANOVAs were performed to examine if there were significant differences in the interaction with online activities and academic success (for research question 2a). The second hierarchical analysis used the mean scores of interactions with the six online activities to cluster students. Similarly, based on cluster membership, one-way ANOVAs were conducted to investigate if students differed in their responses to the self-efficacy, intrinsic goals, and anxiety scales in the self-reported questionnaire (for research question 2b). Because of the small sample size, we also conducted power analyses for all the one-way ANOVAs. To provide an answer to research question 3—the consistency between theory-driven and data-driven approaches—cross-tabulation using students’ clusters resulted from the two above-mentioned hierarchical cluster analyses.

## Results

### Results of research question 1: The joint contributions of self-reported and observational measures to academic success

The results of the correlation analyses are presented in Table 1, which shows that all three scales were significantly associated with the final exam. While both self-efficacy (*r* = 0.37, *p* < 0.01) and intrinsic goals (*r* = 0.49, *p* < 0.01) showed positive relations with the final exam, anxiety was negatively correlated with the final exam (*r* = −0.31, *p* < 0.05). The final exam also had significant and positive relations with all the observed students’ interactions with the online learning activities (*r*s ranging from.27 to.59), except for questions. The variables which had significant correlations with academic success were used as independent variables in the hierarchical regression analyses. Both the values of tolerance (>0.20) and the variance inflation factor (< 5) indicated that there was no presence of multicollinearity. The results of the Koenker test also met the homoscedasticity assumption that supposes that the residuals for the regression model have the same variability along the regression line.

**Table 1:** Results of correlation analyses.

Variables	Self-efficacy	Intrinsic goals	Anxiety	Final exam
*Self-reported measures*				
Self-efficacy	–	–	–	0.37^**^
Intrinsic goals	0.12	–	–	0.49^**^
Anxiety	−0.21	−0.21	–	−0.31^*^
*Observational measures*				
Web pages	0.17	0.40^**^	−0.26	0.27^*^
Videos	−0.02	0.19	−0.28^*^	0.50^**^
Materials	0.43^**^	0.19	−0.24	0.33^*^
Instructions	0.31^*^	0.16	−0.17	0.55^**^
Tests	0.19	0.30^*^	−0.15	0.59^**^
Questions	0.04	−0.01	−0.11	0.06

* *p* < 0.05 and

** *p* < 0.01.

Table 2 presents the results of the hierarchical regression analyses. In regression model 1, where academic success was only regressed on the three scales from the questionnaire, both self-efficacy (*β* = 0.29, *p* < 0.05) and intrinsic goals (*β* = 0.43, *p* < 0.01) made significant contributions to the final exam scores, but anxiety did not (*β* = −0.16, *p* = 0.18). The two scales explained 33% of the variance in academic success: *F* (3, 50) = 9.67, *p* < 0.01, *f* ^2^ = 0.49. In the second regression model, adding the observed students’ interactions with the online learning activities into the regression equation explained an additional 38% of the variance in students’ academic success: *F* (8, 45) = 17.09, *p* < 0.01, *f* ^2^ = 2.45. This means that a combination of students’ self-reported and observational measures of students’ self-regulated learning could explain 71% of the variance in their final exams. Specifically, two of the self-reported scales, self-efficacy (β = 0.35, *p* < 0.01) and intrinsic goals (β = 0.35, *p* < 0.01), still made significant contributions to academic success. Three out of six observed students’ interactions with the online learning activities were significant contributors to academic success: videos (β = 0.54, *p* < 0.01), materials (β = −0.51, *p* < 0.01), and instructions (β = 0.75, *p* < 0.01).

**Table 2:** Results of hierarchical regression analyses.

Variable	*B*	SE	*β*	*t*	Adjusted *R^2^*	*p*	*f* ^2^
*Model 1*					0.33^**^		0.49
Self-efficacy	0.22	0.09	0.29	2.51		0.02	
Intrinsic goals	0.28	0.08	0.43	3.68		0.00	
Anxiety	−0.10	0.07	−0.16	−1.35		0.18	
*Model 2*					0.71^**^		0.41
Self-efficacy	0.26	0.06	0.35	4.07		0.00	
Intrinsic goals	0.23	0.05	0.35	4.21		0.00	
Anxiety	−0.01	0.05	−0.02	−0.23		0.82	
Web pages	0.00	0.00	0.04	0.40		0.69	
Videos	0.03	0.01	0.54	5.63		0.00	
Materials	−0.04	0.01	−0.51	−3.80		0.00	
Instructions	0.08	0.02	0.75	4.45		0.00	
Tests	−0.04	0.04	−0.14	−0.91		0.37	

** *p* < 0.01.

### Results of research question 2a: The differences in the observed students’ interactions with online learning activities and academic success based on the self-reported questionnaire

The hierarchical cluster analysis identified a two-cluster solution of 33 and 21 students in each cluster, respectively (see Table 3). The one-way ANOVAs showed significant differences on all the three self-reported scales between the two clusters: self-efficacy: *F* (1, 52) = 23.70, *p* < 0.01, η^2^ = 0.31; intrinsic goals: *F* (1, 52) = 57.19, *p* < 0.01, η^2^ = 0.52; and anxiety: *F* (1, 52) = 17.49, *p* < 0.01, η^2^ = 0.25. Cluster 1 students reported having higher self-efficacy, a higher level of intrinsic goals, and lower anxiety than cluster 2 students; hence, cluster 1 students were referred to as better self-regulated learners, whereas cluster 2 students were poorer self-regulated learners. The one-way ANOVAs also found that of students’ interactions with the six online learning activities, four of them differed significantly: interactions with web pages: *F*(1, 52) = 4.61, *p* < 0.05, *η*^2^ = 0.08; interactions with videos: *F*(1, 52) = 9.27, *p* < 0.01; *η*^2^ = 0.15; interactions with materials: *F*(1, 52) = 12.56, *p* < 0.01, *η*^2^ = 0.20; and instructions: *F*(1, 52) = 5.07, *p* < 0.05, *η*^2^ = 0.09. So did the final exam scores: *F*(1, 52) = 17.30, *p* < 0.01, *η*^2^ = 0.25. The *M*s showed that the better self-regulated learners interacted with these four online learning activities significantly more frequently, and also obtained higher final exam grades than the poorer self-regulated students. The power analysis results of all the significant results were above the commonly accepted 0.80, except for frequencies of students’ interaction with web pages (0.56) and with instructions (0.60), which also showed small effect sizes (*η*^2^ = 0.08 and *η*^2^ = 0.09 for web pages and instructions, respectively). This suggests that in order for large effect sizes to be detected for the differences on interactions with web pages and instructions with 80% of power and with alpha at 0.05, large sample sizes are needed.

**Table 3:** Hierarchical cluster analysis using the self-reported questionnaire.

Variables	1: Better self-regulated learners (*n* = 33) *M* (SD)	2: Poorer self-regulated learners (*n* = 21) *M* (SD)	*F*	*p*	*η* ^2^	Observed power
*Self-reported measures*						
Self-efficacy	5.39 (0.95)	4.19 (0.77)	23.70	0.00	0.31	1.00
Intrinsic goals	4.67 (1.14)	3.43 (0.93)	57.19	0.00	0.52	1.00
Anxiety	3.18 (0.83)	5.10 (1.01)	17.49	0.00	0.25	0.98
*Observational measures*						
Web pages	508.09 (135.57)	437.00 (84.70)	4.61	0.04	0.08	0.56
Videos	41.00 (10.76)	28.57 (19.26)	9.27	0.00	0.15	0.85
Materials	18.64 (10.38)	10.00 (5.08)	12.56	0.00	0.20	0.94
Instructions	5.45 (9.22)	0.86 (1.77)	5.07	0.03	0.09	0.60
Tests	4.09 (2.51)	2.71 (3.13)	3.19	0.08	0.06	0.42
Questions	24.09 (9.33)	25.00 (7.69)	0.14	0.71	0.00	0.07
*Academic success*						
Final exam	2.71 (0.72)	1.91 (0.68)	17.29	0.00	0.25	0.98

Taken together, better self-regulated learners, who showed the characteristics of having a higher level of self-efficacy and intrinsic goals, and a lower level of anxiety, also tended to browse web page links, watch video lessons, read course learning materials, and view the guidance about the course site significantly more frequently than the poorer self-regulated learners, who reported being less confident, being less intrinsically motivated to learn the course, and having higher anxiety.

### Results of research question 2b: The differences in the self-reported measures of students’ self-regulated learning and academic success based on their observed interactions with online learning activities

The hierarchical cluster analysis using observational measures also produced a two-cluster solution, presented in Table 4. Clusters 1 and 2 had 33 and 12 students, respectively. The one-way ANOVAs found significant differences between the two clusters on all the six observational measures: interactions with web pages: *F*(1, 52) = 74.62, *p* < 0.01, *η*^2^ = 0.59; interactions with videos: *F*(1, 52) = 30.56, *p* < 0.01 *η*^2^ = 0.37; interactions with materials: *F*(1, 52) = 30.54, *p* < 0.01, *η*^2^ = 0.37; interactions with instructions: *F*(1, 52) = 5.65, *p* < 0.01, *η*^2^ = 0.09; interactions with tests: *F*(1, 52) = 10.11, *p* < 0.01, *η*^2^ = 0.16; and interactions with pre-lecture questions: *F*(1, 52) = 13.41, *p* < 0.01, *η*^2^ = 0.21. Specifically, cluster 1 students had a higher frequency of interaction on these four observational measures than their counterparts in cluster 2. Hence, cluster 1 and cluster 2 were referred to as learners with higher and lower online engagement, respectively. On the basis of cluster membership, the one-way ANOVAs further revealed that the two clusters of students also differed significantly in self-reported self-efficacy: *F*(1, 52) = 4.08, *p* < 0.05, *η*^2^ = 0.07 and anxiety: *F*(1, 52) = 16.25, *p* < 0.05, *η*^2^ = 0.24. Cluster 1 students reported having higher self-efficacy and lower anxiety than cluster 2 students did. At the same time, the two clusters of students also differed significantly on their final exam results: *F*(1, 52) = 17.30, *p* < 0.01, *η*^2^ = 0.25. Students with higher online engagement obtained higher final exam grades than their lower-engaged peers. The power analysis results of all the significant results were above the commonly accepted 0.80, except for frequencies of students’ interaction with instructions (0.65) and their self-reported self-efficacy (0.51). The differences between the two clusters on interactions with instructions (*η*^2^ = 0.10) and self-reported self-efficacy (*η*^2^ = 0.07) also had small effect sizes. This means that in order for large effect sizes to be detected for the differences on interactions with instructions and self-efficacy with 80% of power and with alpha at 0.05, large sample sizes are needed. These results demonstrate that the learners with high online engagement not only participated in online learning more frequently than the learners with low online engagement, but they also tended to have a higher level of self-efficacy and a lower level of anxiety, and achieved better academic performance than the learners with low online engagement.

**Table 4:** Hierarchical cluster analysis using the observed students’ interactions with online learning activities.

Variables	1: Higher online engagement (*n* = 33) *M* (SD)	2: Lower online engagement (*n* = 12) *M* (SD)	*F*	*p*	*η* ^2^	Observed power
*Observational measures*						
Web pages	554.82 (94.60)	363.57 (45.15)	74.62	0.00	0.59	1.00
Videos	43.73 (12.22)	24.29 (13.19)	30.56	0.01	0.37	1.00
Materials	19.91 (9.17)	8.00 (4.52)	30.54	0.00	0.37	1.00
Instructions	5.55 (9.21)	0.71 (1.42)	5.65	0.02	0.10	0.65
Tests	4.45 (3.01)	2.14 (1.77)	10.11	0.00	0.16	0.88
Questions	27.55 (7.76)	19.57 (7.86)	13.41	0.00	0.21	0.95
*Self-reported measures*						
Self-efficacy	5.15 (1.10)	4.57 (0.91)	4.08	0.04	0.07	0.51
Intrinsic goals	4.42 (1.03)	3.81 (1.40)	3.44	0.07	0.06	0.46
Anxiety	4.09 (1.10)	5.38 (1.22)	16.25	0.04	0.24	0.98
*Academic success*						
Final exam	4.09 (0.68)	3.29 (0.72)	17.30	0.00	0.25	0.98

### Results of research question 3: Consistency between theory-driven and data-driven approaches

The results of the cross-tabulation analysis revealed a significant and strong association between the cluster membership results from the theory-driven and data-driven approaches: *χ*^2^(1) = 15.31, *p* < 0.01, *φ* = 0.53. Table 5 shows that among students who self-reported as better self-regulated learners, a significantly higher proportion of students were observed as having higher online engagement (81.8%) than having lower online engagement (18.2%). In contrast, of the students who self-reported as poorer self-regulated learners, a significantly higher proportion were observed as having lower online engagement (71.4%) than having higher online engagement (28.6%).

**Table 5:** Results of the cross-tabulation.

Clusters	Count % within online engagement	Higher online engagement	Lower online engagement	Total
Better self-regulated learners	Count	27_a_	6_b_	33
	% within online engagement	81.8%	18.2%	100.0%
Poorer self-regulated learners	Count	6_a_	15_b_	21
	% within online engagement	28.6%	71.4%	100.0%
Total	Count	33	21	54
	% within online engagement	61.1%	38.9%	100.0%

Different subscript letters denote a subset of self-reported categories whose column proportions differ significantly from each other at the 0.05 level.

## Discussion

### Joint contributions of self-reported and observational measures to academic success

The current study combined theory-driven and data-driven approaches to investigate: (1) the joint contributions of students’ self-reported self-efficacy, intrinsic goals and anxiety, and students’ observed interaction with online learning activities to their academic success; and (2) the consistency between theory-driven and data-driven approaches.

Our findings that students’ reporting about their self-efficacy and anxiety significantly contributed to their final exam results corroborated previous findings (Romero-Zaldivar et al., 2012; Joksimović et al., 2015). Similar to previous research (Pardo et al., 2016a,b), we also found that self-efficacy positively affected students’ learning achievement. Different from Pardo et al.’s (2016a) findings that students’ feelings of anxiety during course learning were a significant contributor to their academic success, our study found that only the positive aspects of self-regulated learning (i.e., self-efficacy and intrinsic goals) made significant contributions to their academic success. This difference could possibly be due to the year of the course offering and the size of the course differing between the two studies. The learning context in Pardo et al., was a relatively large compulsory course for first-year students. Therefore, it may have been more important for students to pass the course, because failing the course was highly likely to prevent them from progressing to more advanced courses in their second year of study, and they might face the challenge of being forced to change majors. Hence, anxiety was an important factor in students’ learning. The blended course being examined in the current study was a second-year course and was much smaller in size, which might not impose much peer pressure in terms of succeeding in the course, the result of which being that students might not experience much anxiety in facing the assessments and exams.

Furthermore, consistent with previous findings (Pardo et al., 2016a; Ye and Pennisi, 2022), we also found that positive contributions made by the observational indicators to academic success, which suggested that the more frequently students participated in these online learning activities, the more likely it was that they achieved better learning outcomes, also found positive relations between the observed students’ online learning behaviors and their course marks. However, it should be noted that not all the online learning activities contributed equally to the course marks. Such results seem to suggest that the frequency of learning behaviors alone (quantity) was not able to represent the strategic nature of learning behaviors (quality). Students’ strategic considerations as to how much time and effort should be spent on different learning activities might be better indicators of their academic achievement. As we did not measure the duration of students’ interaction with the online learning activities, such speculation should be examined in future research.

Furthermore, our study showed that the observational measures of the students’ interactions with the online learning activities made a substantially larger contribution than the self-reported data, and explained an extra 38% of the variance in students’ learning outcomes. A combination of the self-reported and the observational data explained around 71% of the variance. This result seems to indicate that our observational measures captured the important elements in online learning that could influence students’ academic performance. Ye and Pennisi (2022) also reported a much larger percentage made by observational data (73%) to students’ academic performance than by self-reported data (11%). However, Ye and Pennisi did not put both self-reported and observational data in the same multiple regression equation; hence, it was unknown whether observational data would still explain the same high percentage of variance in students’ academic performance when taking account of self-reported data.

### Alignment between self-reported and observational measures of students’ self-regulated learning

Whether departing from self-reported measures or departing from observational measures, we found alignment between self-reported and observational measures of students’ self-regulated learning. The cluster analysis identified groups of students who showed similar self-regulated learning experience in terms of how they reported their efficacy, intrinsic goals, and feelings of anxiety, how they learned online by observation, and how well they performed in the course. Within each group, there was coherence between self-reported learning experience and their actual online engagement in the compulsory online part of the course. Students who self-reported having higher efficacy, setting higher intrinsic goals, and feeling less anxious in the course were more likely to be more engaged in online learning—accessing the web pages provided on the course site, viewing course videos, and reading course materials and instructions—significantly more frequently than those who reported lower efficacy, lower intrinsic goals, and more anxiety. At the same time, the better self-regulated learners measured by self-reporting also scored significantly higher on the final exam than the poorer self-regulated learners. These results were consistent with previous findings which reported that self-regulated learners also tended to achieve better academic performance (Kizilcec et al., 2013, 2017).

Furthermore, the positive association between students’ profiles in self-reported measures and observational measures in our cross-tabulation analyses confirmed these alignments; among better self-regulated learners by self-reporting, the majority (81.8%) had higher online engagement by observation, whereas among poorer self-regulated learners by self-reporting, a large proportion of them (71.4%) demonstrated lower online engagement. However, the clustering of the students by the two types of data did not yield perfect overlapping. Such result also corroborated previous research (Pardo et al., 2016a; Li et al., 2020; Ye and Pennisi, 2022) that the alignment between self-reported and observational evidence was only to a certain degree.

The coherence of the evidence collected from different measures and data sources not only served as a way of triangulation, but also strengthened the power of analysis. Combining theory-driven and data-driven approaches enabled presentation of a more comprehensive picture of students’ self-regulated learning experience in blended course designs than could be presented by either self-reported measures or observational measures alone, showing an advantage of a combined approach.

### Implications for teaching practice

As our studies show that students’ self-efficacy and intrinsic goals as well as their frequency of interaction with online learning activities significantly contributed to their academic success, strategies which can boost these elements will be promising for improving students’ learning outcomes. For instance, to empower learners, teachers can provide them with timely feedback as to their learning performance in order to assure them about their competence and to enhance their self-efficacy. The identification of better self-regulated learners early in a course could allow teachers to invite those learners to share their experience, such as how they direct their actions and efforts toward achieving their learning goals, so that those poorer self-regulated learners can make adjustments to emulate their peers. Due to the substantial contributions of online learning to the final exam, teachers should boost students’ motivation to spend time and effort on online learning. For example, instead of leaving students to explore the various functions and sections in the LMS, it may be more desirable for the teaching staff to spend some time in the first couple of weeks of the course to guide students in navigating the course sites. Time should also be spent explicitly explaining the purposes of different online activities, resources, and materials, and how these are linked with learning and teaching in the face-to-face lectures. These strategies which aim to orient students toward online learning and the online course site are likely to encourage students’ active online participation.

### Limitations and directions for future research

Some of the limitations of the study should be taken into consideration when interpreting the results and designing further research along this line of inquiry. First, a major limitation of the study was using frequency of interaction with online learning activities alone to represent students’ online learning engagement. Due to limited analytics functions released by the participants’ university, only frequencies of students’ online interactions were collected. However, frequency is only one of the possible indicators of students’ online learning engagement. In addition to frequency, duration of time spent on different online learning activities and patterns of time-stamped sequences of online learning activities are both possible indicators of students’ online learning engagement (Hadwin et al., 2007; Winters et al., 2008; Bannert et al., 2014; Han et al., 2022). Therefore, future studies should use a combination of observational measures to assess students’ online engagement (Jovanović et al., 2017; Fincham et al., 2019).

Second, the observational measures of students’ interaction with online activities only reflected students’ learning experience in the online part of the whole course, whereas the self-reported measures of students’ self-efficacy, intrinsic goals, and anxiety were concerned with learning in both the face-to-face and online components of the course. Future research should add additional observational measures of students’ engagement in the face-to-face part of learning in blended course designs.

Third, the study was undertaken in a relatively small-sized blended course. Therefore, some of the significant results found in the study had small effect sizes and weak power. However, this reflected the state of learning in blended course designs in the Czech Republic’s higher education sector, especially in the social sciences. Future research in the context of the Czech Republic should be aimed at investigating courses with a large enrolment. Despite the listed limitations, this study is, to the best of our knowledge, the first attempt to combine the self-reported and observational measures to examine students’ self-regulated learning in blended course designs in the Czech Republic.

## Data availability statement

The raw data supporting the conclusions of this article will be made available by the authors, without undue reservation.

## Ethics statement

The studies involving human participants were reviewed and approved by the Ethics Committee of Tomas Bata University in Zlín. The patients/participants provided their written informed consent to participate in this study.

## Author contributions

FH and JV contributed substantially to the conception of the work, analysis, and interpretation of the data. FH, JV, and KJ drafted the work and revised it critically for important intellectual content, approved the final version of the paper to be published, agreed to be accountable for all aspects of the work in ensuring that questions related to the accuracy or integrity of any part of the work are appropriately investigated and resolved. All authors contributed to the article and approved the submitted version.

## Funding

This study was supported by the International Mobilities for Research Activities of the University of Hradec Králové II, CZ.02.2.69/0.0/0.0/18_053/0017841, and by the Faculty of Education, the University of Hradec Králové as part of the project: Individual and contextual predictors of the effect of supporting academic autonomy in future teachers (number: 2101).

## Conflict of interest

The authors declare that the research was conducted in the absence of any commercial or financial relationships that could be construed as a potential conflict of interest.

## Publisher’s note

All claims expressed in this article are solely those of the authors and do not necessarily represent those of their affiliated organizations, or those of the publisher, the editors and the reviewers. Any product that may be evaluated in this article, or claim that may be made by its manufacturer, is not guaranteed or endorsed by the publisher.

## References

[ref1] Ainley M. Patrick L. (2006). Measuring self-regulated learning processes through tracking patterns of student interaction with achievement activities. Educ. Psychol. Rev. 18, 267–286. doi: 10.1007/s10648-006-9018-z

[ref73] Azevedo R. Moos D. C. Greene J. A. Winters F. I. Cromley J. G. (2008). Why is externally-facilitated regulated learning more effective than self-regulated learning with hypermedia? Educational Technology Research and Development, 56, 45–72.

[ref2] Baker R. S. J. Siemens G. (2016). Educational data mining and learning analytics. New York, NY: Cambridge University. 253–272.

[ref4] Bannert M. Reimann P. Sonnenberg C. (2014). Process mining techniques for analysing patterns and strategies in students’ self-regulated learning. Metacogn. Learn. 9, 161–185. doi: 10.1007/s11409-013-9107-6

[ref5] Barnard-Brak L. Lan W. Y. Paton V. O. (2011). “Measuring and profiling self-regulated learning in the online environment” in Fostering Self-regulated Learning Through ICT, eds. G. Dettori and D. Persico (Pennsylvania: IGI Global), 27–38.

[ref6] Beishuizen J. Steffens K. (2011). “A conceptual framework for research on self-regulated learning” in Self-regulated Learning in Technology Enhanced Learning Environments, eds. R. Carneiro and P. Lefrere (Leiden:Brill), 1–19.

[ref7] Bettinger E. P. Baker R. B. (2014). The effects of student coaching: an evaluation of a randomized experiment in student advising. Educ. Eval. Policy Anal. 36, 3–19. doi: 10.3102/0162373713500523

[ref74] Boekaerts M. Cascallar E. (2006). How far have we moved toward the integration of theory and practice in self-regulation? Educational psychology review 18, 199–210.

[ref8] Broadbent J. Fuller-Tyszkiewicz M. (2018). Profiles in self-regulated learning and their correlates for online and blended learning students. Educ. Technol. Res. Dev. 66, 1435–1455. doi: 10.1007/s11423-018-9595-9

[ref9] Broadbent J. Poon W. L. (2015). Self-regulated learning strategies & academic achievement in online higher education learning environments: a systematic review. Internet High. Educ. 27, 1–13. doi: 10.1016/j.iheduc.2015.04.007

[ref10] Buckingham Shum S. Crick R. D. (2012). Learning dispositions and transferable competencies: pedagogy, modelling and learning analytics.in *Proceedings of the 2nd International Conference on Learning Analytics and Knowledge* (Vancouver British Columbia Canada: ACM), 92–101.

[ref11] Chen B. Resendes M. Chai C. S. Hong H.-Y. (2017). Two tales of time: uncovering the significance of sequential patterns among contribution types in knowledge-building discourse. Interact. Learn. Environ. 25, 162–175. doi: 10.1080/10494820.2016.1276081

[ref12] Dabbagh N. Kitsantas A. (2013). “Using learning management systems as metacognitive tools to support self-regulation in higher education contexts” in International Handbook of Metacognition and Learning Technologies (Berlin: Springer), 197–211.

[ref13] Dent A. L. Koenka A. C. (2016). The relation between self-regulated learning and academic achievement across childhood and adolescence: a meta-analysis. Educ. Psychol. Rev. 28, 425–474. doi: 10.1007/s10648-015-9320-8

[ref14] Duncan T. G. McKeachie W. J. (2005). The making of the motivated strategies for learning questionnaire. Educ. Psychol. 40, 117–128. doi: 10.1207/s15326985ep4002_6

[ref15] Fincham E. Gasevic D. Jovanovic J. Pardo A. (2019). From study tactics to learning strategies: an analytical method for extracting interpretable representations. IEEE Trans. Learning Technol. 12, 59–72. doi: 10.1109/TLT.2018.2823317

[ref16] Gašević D. Dawson S. Siemens G. (2015). Let’s not forget: learning analytics are about learning. Tech. Trends 59, 64–71. doi: 10.1007/s11528-014-0822-x

[ref17] Gibson A. Aitken A. Sándor Á. Buckingham Shum S. Tsingos-Lucas C. Knight S. (2017). Reflective writing analytics for actionable feedback. in *Proceedings of the Seventh International Learning Analytics and Knowledge Conference*, 153–162.

[ref18] Greene J. A. Azevedo R. (2010). The measurement of learners’ self-regulated cognitive and metacognitive processes while using computer-based learning environments. Educ. Psychol. 45, 203–209. doi: 10.1080/00461520.2010.515935

[ref19] Hadwin A. F. Nesbit J. C. Jamieson-Noel D. Code J. Winne P. H. (2007). Examining trace data to explore self-regulated learning. Metacogn. Learn. 2, 107–124. doi: 10.1007/s11409-007-9016-7

[ref20] Han F. (2022). Recent development in university student learning research in blended course designs: combining theory-driven and data-driven approaches. Front. Psychol. 13. doi: 10.3389/fpsyg.2022.905592 PMC9152441 35656502

[ref21] Han F. Ellis R. A. (2021). Predicting students’ academic performance by their online learning patterns in a blended course. Educ. Technol. Soc. 24, 191–204.

[ref22] Han F. Ellis R. A. Pardo A. (2022). The descriptive features and quantitative aspects of students observed online learning: how are they related to self-reported perceptions and learning outcomes. IEEE Trans. Learn. 15, 32–41. doi: 10.1109/TLT.2022.3153001

[ref23] Jakesova J. Hrbackova K. (2014). The Czech adaptation of motivated strategies for learning questionnaire (MSLQ). ASS 10:p72. doi: 10.5539/ass.v10n12p72

[ref24] Järvelä S. Hadwin A. F. (2013). New frontiers: regulating learning in CSCL. Educ. Psychol. 48, 25–39. doi: 10.1080/00461520.2012.748006

[ref25] Jarvoll A. B. (2018). “I’ll have everything in diamonds!”: students’ experiences with Minecraft at school. Stud. Paedagog. 23, 67–69. doi: 10.5817/SP2018-4-4

[ref26] Joksimović S. Gašević D. Kovanović V. Riecke B. E. Hatala M. (2015). Social presence in online discussions as a process predictor of academic performance. J. Comput. Assist. Learn. 31, 638–654. doi: 10.1111/jcal.12107

[ref27] Jovanović J. Gašević D. Dawson S. Pardo A. Mirriahi N. (2017). Learning analytics to unveil learning strategies in a flipped classroom. Internet High. Educ. 33, 74–85. doi: 10.1016/j.iheduc.2017.02.001

[ref28] Kaendler C. Wiedmann M. Rummel N. Spada H. (2015). Teacher competencies for the implementation of collaborative learning in the classroom: a framework and research review. Educ. Psychol. Rev. 27, 505–536. doi: 10.1007/s10648-014-9288-9

[ref75] Karanicolas S. Snelling C. Winning T. Chapman J. Williams A. Douglas T. (2018). Translating concept into practice: enabling first-year health sciences teachers to blueprint effective flipped learning approaches: final report. Canberra, ACT: Department of Education and Training.

[ref29] Kizilcec R. F. Pérez-Sanagustín M. Maldonado J. J. (2017). Self-regulated learning strategies predict learner behavior and goal attainment in massive open online courses. Comput. Educ. 104, 18–33. doi: 10.1016/j.compedu.2016.10.001

[ref30] Kizilcec R. F. Piech C. Schneider E. (2013). Deconstructing disengagement: analyzing learner subpopulations in massive open online courses. in *Proceedings of the Third International Conference on Learning Analytics and Knowledge*, 170–179.

[ref31] Krumm A. E. Waddington R. J. Teasley S. D. Lonn S. (2014). “A learning management system-based early warning system for academic advising in undergraduate engineering” in Learning Analytics, eds. J. Larusson and B. White (Berlin: Springer), 103–119.

[ref32] Li Q. Baker R. Warschauer M. (2020). Using clickstream data to measure, understand, and support self-regulated learning in online courses. Internet High. Educ. 45:100727. doi: 10.1016/j.iheduc.2020.100727

[ref33] Lockyer L. Heathcote E. Dawson S. (2013). Informing pedagogical action: aligning learning analytics with learning design. Am. Behav. Sci. 57, 1439–1459. doi: 10.1177/0002764213479367

[ref34] Lynn L. N. Cuskelly M. O’Callaghan M. J. Gray P. H. (2011). Self-regulation: a new perspective on learning problems experienced by children born extremely preterm. Aust. J. Educ. Dev. Psychol. 11, 1–10.

[ref35] Martinez-Maldonado R. Hernandez-Leo D. Pardo A. Suthers D. Kitto K. Charleer S.. (2016). Cross-LAK: learning analytics across physical and digital spaces. in *Proceedings of the Sixth International Conference on Learning Analytics & Knowledge*, 486–487.

[ref36] McCaslin M. Hickey D. T. (2001). Educational psychology, social constructivism, and educational practice: a case of emergent identity. Educ. Psychol. 36, 133–140. doi: 10.1207/S15326985EP3602_8

[ref37] Moos D. C. Stewart C. A. (2013). “Self-regulated learning with hypermedia: bringing motivation into the conversation” in International Handbook of Metacognition and Learning Technologies, eds. R. Azevedo and V. Aleven (Berlin: Springer), 683–695.

[ref38] Ocumpaugh J. Baker R. Gowda S. Heffernan N. Heffernan C. (2014). Population validity for educational data mining models: a case study in affect detection. Br. J. Educ. Technol. 45, 487–501. doi: 10.1111/bjet.12156

[ref39] Pardo A. Han F. Ellis R. A. (2016a). Combining university student self-regulated learning indicators and engagement with online learning events to predict academic performance. IEEE Trans. Learn. Technol. 10, 82–92. doi: 10.1109/TLT.2016.2639508

[ref40] Pardo A. Han F. Ellis R. A. (2016b). Exploring the relation between self-regulation, online activities, and academic performance: a case study. in *Proceedings of the SIXth International Conference on Learning Analytics and Knowledge*, 422–429.

[ref41] Pintrich P. R. (2000). “The role of goal orientation in self-regulated learning” in Handbook of Self-regulation (Amsterdam: Elsevier), 451–502.

[ref42] Pintrich P. R. De Groot E. V. (1991). Motivated strategies for learning questionnaire. J. Educ. Psychol.

[ref43] Pintrich P. R. Zusho A. (2002). “Student motivation and self-regulated learning in the college classroom” in Higher Education: Handbook of Theory and Research (Amsterdam: Springer), 55–128.

[ref44] Reimann P. Markauskaite L. Bannert M. (2014). E-research and learning theory: what do sequence and process mining methods contribute?: e-research and learning theory. Br. J. Educ. Technol. 45, 528–540. doi: 10.1111/bjet.12146

[ref45] Richardson J. T. (2017). Student learning in higher education: a commentary. Educ. Psychol. Rev. 29, 353–362. doi: 10.1007/s10648-017-9410-x

[ref46] Rienties B. Toetenel L. (2016). The impact of learning design on student behaviour, satisfaction and performance: a cross-institutional comparison across 151 modules. Comput. Hum. Behav. 60, 333–341. doi: 10.1016/j.chb.2016.02.074

[ref47] Rodríguez-Triana M. J. Martínez-Monés A. Asensio-Pérez J. I. Dimitriadis Y. (2015). Scripting and monitoring meet each other: aligning learning analytics and learning design to support teachers in orchestrating CSCL situations: scripting and monitoring meet each other. Br. J. Educ. Technol. 46, 330–343. doi: 10.1111/bjet.12198

[ref48] Romero C. López M.-I. Luna J.-M. Ventura S. (2013). Predicting students’ final performance from participation in on-line discussion forums. Comput. Educ. 68, 458–472. doi: 10.1016/j.compedu.2013.06.009

[ref49] Romero-Zaldivar V.-A. Pardo A. Burgos D. Kloos C. D. (2012). Monitoring student progress using virtual appliances: a case study. Comput. Educ. 58, 1058–1067. doi: 10.1016/j.compedu.2011.12.003

[ref50] Sclater N. Peasgood A. Mullan J. (2016). Learning Analytics in Higher Education. London: JISC.

[ref51] Shin Y. Park J. Lee S. (2018). Improving the integrated experience of in-class activities and fine-grained data collection for analysis in a blended learning class. Interact. Learn. Environ. 26, 597–612. doi: 10.1080/10494820.2017.1374980

[ref52] Siemens G. (2013). Learning analytics: the emergence of a discipline. Am. Behav. Sci. 57, 1380–1400. doi: 10.1177/0002764213498851

[ref53] Sun Z. Xie K. Anderman L. H. (2018). The role of self-regulated learning in students’ success in flipped undergraduate math courses. Internet High. Educ. 36, 41–53. doi: 10.1016/j.iheduc.2017.09.003

[ref54] Tang Y. M. Chen P. C. Law K. M. Wu C.-H. Lau Y. Guan J.. (2021). Comparative analysis of Student’s live online learning readiness during the coronavirus (COVID-19) pandemic in the higher education sector. Comput. Educ. 168:104211. doi: 10.1016/j.compedu.2021.104211, PMID: 33879955 PMC8049721

[ref56] Vanslambrouck S. Zhu C. Pynoo B. Lombaerts K. Tondeur J. Scherer R. (2019). A latent profile analysis of adult students’ online self-regulation in blended learning environments. Comput. Hum. Behav. 99, 126–136. doi: 10.1016/j.chb.2019.05.021

[ref57] Venkatesh V. Shaikh K. Zuberi A. Urbaniak K. Gallant T. Lakhana A. (2013). “Development of task understanding and monitoring in information retrieval environments: demystifying metacognitive and self-regulatory mechanisms in graduate learners using topic maps indexing technologies to improve essay-writing skills” in International Handbook of Metacognition and Learning Technologies (Berlin: Springer), 277–291.

[ref58] Vermunt J. D. Donche V. (2017). A learning patterns perspective on student learning in higher education: state of the art and moving forward. Educ. Psychol. Rev. 29, 269–299. doi: 10.1007/s10648-017-9414-6

[ref59] Winkelmann K. Keeney-Kennicutt W. Fowler D. Lazo Macik M. Perez Guarda P. Joan Ahlborn C. (2020). Learning gains and attitudes of students performing chemistry experiments in an immersive virtual world. Interact. Learn. Environ. 28, 620–634. doi: 10.1080/10494820.2019.1696844

[ref60] Winne P. H. (1996). A metacognitive view of individual differences in self-regulated learning. Learn. Individ. Differ. 8, 327–353. doi: 10.1016/S1041-6080(96)90022-9

[ref61] Winne P. H. (2001). “Self-regulated learning viewed from models of information processing” in Self-Regulated Learning and Academic Achievement: Theoretical Perspectives, eds. B. J. Zimmerman and D. H. Schunk, Routledge. 145.

[ref62] Winne P. H. Hadwin A. F. (1998). “Studying as self-regulated learning” in Metacognition in Educational Theory and Practice. eds. Hacker D. J. Dunlosky J. Graesser A. C., 277–304.

[ref63] Winne P. H. Perry N. E. (2000). “Measuring self-regulated learning,” in Handbook of Self-Regulation, eds. M. Boekaerts, P. R. Pintrich, and M. Zeidner. 531–566. Amsterdam: Elsevier.

[ref64] Winters F. I. Greene J. A. Costich C. M. (2008). Self-regulation of learning within computer-based learning environments: a critical analysis. Educ. Psychol. Rev. 20, 429–444. doi: 10.1007/s10648-008-9080-9

[ref65] Wong R. (2022). Basis psychological needs of students in blended learning. Interact. Learn. Environ. 30, 984–998. doi: 10.1080/10494820.2019.1703010

[ref66] Wong J. Baars M. Davis D. Van Der Zee T. Houben G.-J. Paas F. (2019). Supporting self-regulated learning in online learning environments and MOOCs: a systematic review. Int. J. Hum–Comput. Int. 35, 356–373. doi: 10.1080/10447318.2018.1543084

[ref67] Wong B. T. Li K. C. (2020). A review of learning analytics intervention in higher education (2011–2018). J. Comput. Educ. 7, 7–28. doi: 10.1007/s40692-019-00143-7

[ref68] Ye D. Pennisi S. (2022). Using trace data to enhance students’ self-regulation: a learning analytics perspective. Internet High. Educ. 54:100855. doi: 10.1016/j.iheduc.2022.100855

[ref69] Zhou M. Winne P. H. (2012). Modeling academic achievement by self-reported versus traced goal orientation. Learn. Instr. 22, 413–419. doi: 10.1016/j.learninstruc.2012.03.004

[ref70] Zimmerman B. J. (2008). Investigating self-regulation and motivation: historical background, methodological developments, and future prospects. Am. Educ. Res. J. 45, 166–183. doi: 10.3102/0002831207312909

[ref71] Zimmerman B. J. Schunk D. H. (2001). Self-regulated Learning and Academic Achievement: Theoretical Perspectives. eds. B. J. Zimmerman and D. H. Schunk. London: Routledge.

[ref72] Zimmerman B. J. Schunk D. H. (2011). “Self-regulated learning and performance: an introduction and an overview” in Handbook of Self-regulation of Learning and Performance (Routledge) 15–26.

